# Walking Football as a Multidimensional Intervention for Healthy Aging: A Scoping Review of Physical and Functional Outcomes in Older Adults

**DOI:** 10.3390/ijerph22101533

**Published:** 2025-10-07

**Authors:** Paulo Sérgio Machado Rodrigues, Antônio Ribeiro Neto, Leandro Alonso do Espírito Santo, Sheilla Tribess, Jair Sindra Virtuoso Junior

**Affiliations:** 1 Health Care Graduate Program, Federal University of Triângulo Mineiro, Uberaba 38025-440, MG, Brazil; ps_mrodrigues@hotmail.com (P.S.M.R.); antoniorn11@yahoo.com.br (A.R.N.); 2 Graduate Program in Physical Education, Federal University of Triângulo Mineiro, Uberaba 38061-500, MG, Brazil; leandro22alonso@hotmail.com; 3 Center of Research in Physical Activity and Health, Federal University of Triângulo Mineiro, Uberaba 38061-500, MG, Brazil; sheilla.tribess@uftm.edu.br

**Keywords:** healthy aging, walking football, physical fitness, older adults, health promotion

## Abstract

Population aging presents pressing public health challenges, calling for accessible and effective interventions to preserve functional capacity and prevent frailty. Walking football, an adapted sport for older adults, has emerged as a promising approach by combining aerobic activity, neuromuscular stimulation, and social engagement. This scoping review aimed to synthesize experimental evidence on its effect on physical and functional outcomes in older adults. Following PRISMC-ScR and Joanna Briggs Institute guidelines, a systematic search across seven databases identified 10 studies with 332 participants (mean age 68.7 years, 85.2% male). Interventions lasted 6–16 weeks, with two to three weekly sessions. The outcomes assessed included strength, agility, cardiorespiratory fitness, body composition, and clinical parameters. Six studies reported improvements in at least one component of physical fitness, particularly handgrip strength, abdominal fat reduction, and aerobic capacity. Among the included studies, three were randomized controlled trials, four quasi-experimental designs, and three intervention studies without control groups, highlighting the methodological heterogeneity of the field. Some studies also reported benefits in blood pressure, glucose, cholesterol, and quality of life. Despite promising results, the literature lacks large-scale randomized trials, female representation, and long-term assessments. Walking football appears to be a safe, multicomponent, and low-cost strategy to support healthy aging and warrants integrations into public health policies.

## 1. Introduction

Health promotion, as defined by the World Health Organization (WHO), is a process of enabling individuals and communities to increase control over their health and improve their quality of life [1]. It goes beyond the boundaries of the healthcare sector by incorporating public policies, healthy environments, personal skill development, and the reorientation of health services [2,3,4]. When applied to population aging, the concept of health promotion acquires particular relevance. Beyond extending chronological life expectancy, it highlights the potential to substantially increase healthy life expectancy; that is, the number of years lived free from disability and functional dependence, as recommended by the World Health Organization [5]. This perspective underscores the urgent need for strategies capable of ensuring not only longer lives but also lives lived with autonomy, functionality, and quality.

In this context, the term “significant increase in longevity” refers not only to chronological life expectancy but also to healthy life expectancy; that is, the number of years lived free from disability and functional dependence, as recommended by the WHO.

According to WHO guidelines, older adults should engage in 150–300 min of moderate-intensity aerobic physical activity or 75–150 min of vigorous-intensity aerobic activity per week, in addition to muscle-strengthening activities involving major muscle groups on two or more days [6]. Such evidence consistently indicates that adherence to physical activity guidelines is associated with an increase in healthy life expectancy, reducing morbidity, delaying frailty onset, and improving quality of life in older populations, whilst also serving as a benchmark for evaluating physical activity promotion interventions such as walking football (WF).

Aging is a natural part of the life course and experiencing it in a healthy manner implies the preservation of autonomy, functional capacity, and quality of life, even in the face of physiological, psychological, and social changes inherent to advanced age [7]. In this context, regular physical exercise is one of the most well-documented interventions for promoting health in older adults, acting thorough multiple biological and psychological mechanisms of aging [7,8]. Among its most consistent effects are the maintenance of muscle strength and power, improvement in cardiorespiratory fitness, and prevention of sarcopenia, osteoporosis, and falls [9,10,11]. Moreover, evidence highlights the role of adapted sports such as WF, which combine aerobic, neuromuscular, and social components, as promising alternatives to support functional capacity and engagement in older adults [12].

In this review, we distinguish physical activity (any bodily movement produced by skeletal muscles that results in energy expenditure) from exercise (planned, structured, and repetitive activity aimed at improving or maintaining physical fitness) and from adapted sport (rules and environments modified to ensure safety and accessibility for older adults). Accordingly, WF is treated as an adapted sport that operationalizes structured exercise within a group-based physical activity context; when referring to effects beyond the session structure, we use the umbrella term physical activity.

In this manuscript, WF is conceptualized as an adapted sport that implements structured exercise within a broader physical activity framework; therefore, when reporting outcomes, we explicitly refer to exercise-related adaptations (e.g., strength, aerobic fitness) while using physical activity as the umbrella term when addressing participation and exposure.

WF, an adapted sport modality for individuals aged 50 and over, has gained international attention as an accessible, safe, and highly adherent strategy by combining aerobic exercise, neuromuscular stimulation, and social interaction in regular sessions [8,11,12]. With the potential to induce significant functional adaptations, this practice has emerged as a promising alternative for older adults seeking to remain physically active in structured and group-based contexts.

WF interventions are typically delivered in community clubs, recreational facilities, or health promotion programs, and sessions are commonly facilitated by physical education professionals, specialized coaches, or trained facilitators with experience in adapted sports for older adults [8]. The costs associated with WF are generally low, as the activity makes use of existing sports facilities and requires minimal equipment, which supports its feasibility for public health programs and community initiatives [11]. Moreover, in some contexts, gender-sensitive adaptations have been reported, such as female-only programs, recognizing cultural and psychosocial barriers to participation among women and promoting more equitable access [12].

However, although observational studies and preliminary trials suggest benefits of WF on physical, metabolic, and psychosocial indicators, important gaps remain regarding its efficacy in experimental designs. There is a scarcity of studies with control groups, pre- and post-intervention assessments, and a specific focus on physical fitness components related to functional performance in older adults. Therefore, consolidating the available evidence is essential to inform clinical decision-making, public health programs, and future research.

This study was designed to address these gaps by analyzing the feasibility, safety, and potential health impacts of WF in older adults, structured around three specific objectives. Recent studies have provided additional evidence on feasibility, safety, physiological demands, and health benefits of WF in older populations [13,14,15]. The specific objectives were: (1) to identify experimental studies that have implemented WF in older adults; (2) to characterize the methodological designs, intervention protocols, and outcomes assessed; and (3) to synthesize the main physical and functional effects reported, highlighting gaps and directions for future research.

## 2. Materials and Methods

This scoping review was conducted in accordance with the recommendations of the Joanna Briggs Institute Reviewer’s Manual for scoping reviews [16] and reported following the Preferred Reporting Items for Systematic Reviews and Meta-Analyses extensions for Scoping Reviews (PRISMA-ScR) checklist [17]. This review protocol was previously registered in the International Prospective Register of Systematic Reviews (PROSPERO) under the number CRD420250650932.

### 2.1. Eligibility Criteria

The inclusion criteria were structured based on the Population-Concept-Context (PCC) framework [16]:

1—Population—Studies that included individuals aged 60 years or older, according to the definition of the WHO.

2—Concept—Intervention involving WF, based on the guidelines of the Walking Football Association Organization [18], which included structured activities with walking, passing, and tactical interactions, conducted safely and adapted for older adults. Eligible studies were required to assess at least one outcome related to physical fitness (strength, power, agility or cardiorespiratory endurance), physiological parameters or functional markers.

3—Context—Experimental study designs were included (randomized controlled trials, quasi-experimental and intervention studies without control groups), with no restrictions regarding language, geographic location, or publication date.

### 2.2. Information Sources and Search Strategy

The initial search was conducted in the International Prospective Register of Systematic Reviews (PROSPERO), the Cochrane Database, and the JBI Evidence Synthesis to identify previous or ongoing reviews on the topic.

A systematic search was carried out in the following electronic databases: MEDLINE (via PubMed), SciELO, Scopus, LILACS, EMBASE, SPORTDiscus, and Web of Science. The initial search took place on 10 December 2024 and was updated on 15 January 2025, with no restrictions on language or publication date. In addition, manual searching was performed through screening the reference lists of all included articles and relevant reviews, forward citation tracking, and expert consultation (“snowballing”).

The initial strategy was developed for the MEDLINE database using controlled descriptors (MeSH) and free-text terms, combined using Boolean operators. This strategy was subsequently adapted to each database according to its specific terminology. The search formulation and peer review followed the Peer Review of Electronic Search Strategies (PRESS) guidelines [19]. An example of search terms used: (“Walking Football” [MeSH Terms] OR “Walking Football” [Title/Abstract] OR “Walking Soccer” [Title/Abstract]) AND (“Aged” [MeSH Terms] OR elder* OR “older adults” OR “older people”) AND (“Exercise” [MeSH Terms] OR “physical activity” OR “physical fitness”).

### 2.3. Selection Process

All identified records were initially subjected to manual duplicate removal using the Rayyan software (Rayyan QCRI, version 0.1.0, Doha, Qatar) [20]. Two independent reviewers (PSMR and ARN) screened titles and abstracts based on the eligibility criteria. Potentially eligible studies were then assessed in full text. Discrepancies were resolved by consensus or, when necessary, with the assistance of a third reviewer (JSVJ).

### 2.4. Data Extraction and Analysis

Data extraction was conducted by two independent reviewers using a standardized and pre-tested spreadsheet developed by the authors (PSMR, ARN) and piloted on three randomly selected studies to ensure clarity and consistency. Extraction domains were guided by the JBI manual and organized following PICO. The following information was extracted:

- Study characteristics: authorship, year of publication, country, and study design.
- Population characteristics: sample size, mean age, body mass index (BMI), presence of comorbidities and medication use.
- Intervention details: frequency, duration, and components of the WF protocol, setting (indoor/outdoor), team size, and adherence/dropout rates.
- Outcomes: strength, power, cardiorespiratory endurance, agility, other functional or clinical markers.

## 3. Results

### 3.1. Study Identification and Selection

**Figure 1:**
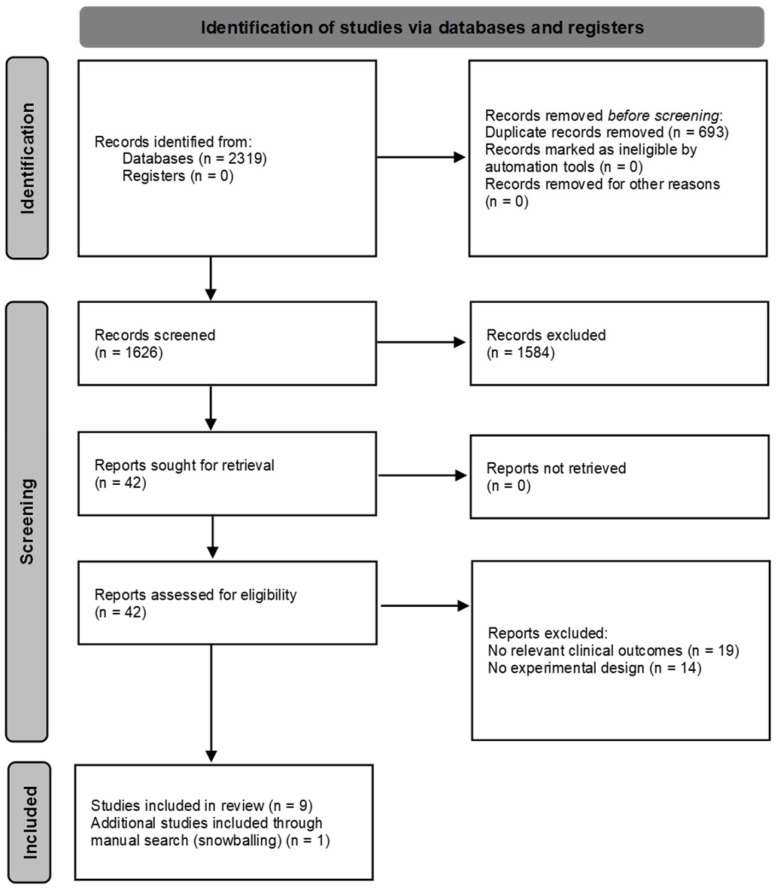
Flowchart diagram of the study selection process.

The systematic search identified 2319 records. After removing duplicates (*n* = 693), 1626 titles and abstracts were screened, resulting in 42 articles assessed in full text. Of these, 33 were excluded for not meeting the eligibility criteria, and one additional study was identified through manual searching, yielding a total of 10 studies included in the final analysis (Figure 1). These studies were conducted in six countries, predominantly in Western Europe.

The body of evidence comprised three randomized controlled trials (RCTs), two quasi-experimental studies, and five observational studies (cross-sectional or longitudinal designs), reflecting both exploratory initiatives and structured interventions evaluating WF in older adults.

Across studies, the mean age ranged from 61 to 73 years, with a predominance of male participants (>80%). Some samples included individuals with chronic conditions such as type 2 diabetes, cardiovascular risk factors, and prostate cancer under androgen deprivation therapy. Socioeconomic data were rarely reported. Intervention protocols lasted 6 to 16 weeks, with two to three sessions per week, each lasting 60–120 min. Sessions were delivered in community clubs, health units, or by local football associations and, in some cases, through the charitable arms of professional football clubs, which provided facilities and organizational support, and facilitated by physical education professionals or specialized coaches. Adherence ranged from 81% to 97%, with dropout rates below 15%. Costs were minimal, as existing facilities and low-cost equipment were used. Settings varied between indoor gymnasiums and outdoor synthetic or grass pitches, and team sizes ranged from 5 to 7 players per side.

Significant improvements were reported in handgrip strength, lower-limb strength, balance, time-to-exhaustion, and cardiorespiratory fitness (VO_2_peak). Reductions in abdominal fat, body fat percentage, and glycemic levels were also observed. Importantly, WF was consistently associated with high enjoyment, adherence, and safety, with only isolated minor injuries reported.

### 3.2. Characteristics of Included Studies

The 10 included studies were published between 2015 and 2024, conducted across six coutries and involved a total of 332 participants with a mean age of 68.7 years. Most participants were male (85.2%) and sample sizes ranged from 10 to 60 individuals per study.

The average duration of the intervetion was 10 weeks, with a frequency of two to three sessions per week. Only three studies inclued a control group. Most adpoted quasi-experimental or observational designs, with low female representation and methodological heterogeneity.

### 3.3. Outcomes Assessed

The outcomes covered domains of physical fitness, body composition and physiological parameters:

- Muscle strength and power: improvements in handgrip strength and vertical jump test.
- Agility and balance: improvements in test such as Time Up and Go and single-leg balance.
- Cardiorespiratory fitness: increases in estimated VO_2_ and time to exhaustion.
- Body composition: reductions in abdominal circumference and body fat percentage.
- Clinical and biochemical parameters: improvements in blood pressure, blood glucose and cholesterol in specific subgroups.
- Psychological aspects: greater enjoyment of the activity and improved quality of life reported in two studies.

Six of the ten studies reported significant improvements in at least one physical or functional marker. In the three studies with a control group, the gains were superior in the intervention group (Table 1).

## 4. Discussion

This scoping review identified and characterized ten experimental studies that evaluated the effects of WF on older adults. The findings demonstrate that this adapted form of exercise promotes improvements in several components of physical fitness and functionality, such as muscle strength, agility, cardiorespiratory capacity and body composition, with good tolerance and adherence potential. These results support existing literature that positions regular physical activity as a key element in promoting healthy aging [7,8].

When compared to other physical activity interventions for older adults, such as walking groups, resistance training, and gym-based structured programs, WF shows comparable or superior adherence rates, high acceptability due to its social and recreational nature, and meaningful improvements in functional outcomes. Unlike structured gym-based programs [10,11], WF provides a community-oriented and enjoyable context, which may support long-term retention. However, compared to walking groups [10,11], or resistance training protocols [25], WF requires adequate supervision and facilities, which may limit scalability in some contexts.

### 4.1. Clinical and Functional Relevance

By combining aerobic stimulation with motor challenges and social interaction, WF constitutes a multicomponent intervention-a characteristic associated with greater effects on physical function and the prevention of frailty syndrome in older adults [25]. The studies included in this review reported gains in handgrip strength, which is widely used in public health and geriatrics as a global indicator of muscle strength and risk of disability [26], although its direct relationship with the specific demands of WF is limited. Additionally, this review observed improvements in time to exhaustion and mobility, reinforcing the positive effects of WF on overall functional capacity. Such outcomes are clinically relevant as they are linked to reduced risk of falls, hospitalizations and functional dependency [10,11]. Reductions in blood pressure and improvements in glycemic control observed in some studies are clinically relevant, as they are directly associated with lower cardiovascular risk and reduced incidence of related complications in older adults [19].

### 4.2. Methodological Gaps and Representativeness

Despite the benefits identified, the literature presents important limitations. Most samples consisted exclusively of men (85.2%), limiting generalizability to women. Previous studies suggest that sociocultural barriers, stigmas, and individual preferences may hinder women’s participation in sports such as football, even in adapted formats [27,28]. Trials exploring strategies for inclusive and culturally sensitive adaptation of the modality for older women are needed.

Furthermore, only 30% of studies included control groups, and the predominance of quasi-experimental designs undermines the inferential robustness of results. The lack of randomized trials and the limited number of studies evaluating physiological or biochemical markers (e.g., blood pressure, glucose, cholesterol) underscore the need for better-structured clinical trials with objective measures and long-term follow-up [29]. Caution is warranted, however, regarding the feasibility and acceptability of RCTs in real-world contexts, where qualitative research can play a complementary role in capturing participants’ experiences and mechanisms of adherence [30,31].

### 4.3. Comorbidities and Polypharmacy

Aging is commonly accompanied by chronic conditions such as type 2 diabetes, hypertension and osteopenia, along with frequent polypharmacy [32,33]. Although six studies reported the presence of comorbidities and only five mentioned medication use, few explored the influence of these conditions on outcomes or the therapeutic effects of the intervention. Previous research suggests that WF may improve glycemic control, blood pressure and lipid profiles, enhancing its potential as an adjunct intervention in primary healthcare setting [34,35,36].

### 4.4. Exercise Intensity and Intervention Components

Most intervention reported moderate to vigorous perceived exertion, with average heart rates around 80% of the estimated maximum, aligning with international recommendations for promoting cardiovascular health in older adults [30]. However, there was a lack of specific evaluations of lower limb strength and power, which are critical for mobility, fall prevention and maintaining functional independence [25].

Regarding body composition, while some studies showed a reduction in fat mass and abdominal circumference, few reported increases in lean mass. This is understandable, given the aerobic nature of activity [37], but suggests that combining WF with resistance training may yield greater benefits in sarcopenic or frail populations [38]. In this regard, combining WF with resistance exercises could optimize improvements in muscle mass, strength, and functional independence, enhancing its overall effectiveness as a health promotion strategy for older adults.

### 4.5. Psychosocial Variable and Adherence

Social interaction and enjoyment-key elements in WF were underexplored in the review studies. Only two studies addressed quality of life and motivation as outcomes. Considering the growing importance of psychosocial factors in healthy aging, future research should integrate qualitative assessments, subjective well-being, and long-term adherence to sports practice [30,31].

### 4.6. Strengths and Limitations of This Study

This review followed a registered protocol and adhered to PRISMA-ScR guidelines, ensuring methodological rigor. It provides a comprehensive synthesis of WF interventions, including diverse outcomes and participant profiles. However, limitations include the small number of available studies, methodological heterogeneity, predominance of male participants, and lack of standardized reporting on costs and socioeconomic indicators. The reference to “low cost,” therefore, should be contextualized as indirectly inferred from study descriptions, since systematic cost assessments were absent. These aspects restrict the generalizability of findings and highlight the need for larger, gender-sensitive, and methodologically robust trials, as well as qualitative investigations to deepen understanding of acceptability and sustainability.

### 4.7. Implications and Future Directions

This review highlights WF as a safe, accessible, and potentially effective intervention for promoting functional health in older adults. However, large-scale randomized clinical trials with objective measures, diverse populations (including women and frail older adults), and comprehensive evaluations of physiological and psychosocial variables are essential. The development of specific clinical guidelines for the application of WF in geriatric practice, including individualized prescriptions, combined protocols, and adherence strategies, could facilitate its integration into health services and active aging programs. Although WF has been described as a low-cost intervention due to the use of existing facilities and minimal equipment, this claim is not yet supported by formal economic analyses. Future studies should investigate cost-effectiveness to substantiate its scalability in public health contexts.

## 5. Conclusions

WF is a safe, low-cost, and potentially effective intervention for promoting functional health in older adults. By integrating aerobic, neuromuscular and social components, this modality shows promise in mitigating physical decline and encouraging social participation in community setting.

Despite the positive results, the evidence remains limited by non-randomized designs, low female inclusion, and lack of long-term assessments. Studies lack methodological standardization and robust analyses of integrated clinical, physiological and psychosocial markers.

It is concluded that WF has relevant translational potential and should be considered in active aging programs, especially in primary healthcare. However, large-scale randomized clinical trials with diverse samples and objective measures are still needed to confirm its effectiveness and support its inclusion in public health policies for older adults.

## Figures and Tables

**Table 1:** Experimental studies on WF in older adults: designs, samples and outcomes assessed.

Study	Country	Participants	Male (%)	Mean Age (Years)	Duration (Weeks)	Setting (Indoor/Outdoor)	Team Size	Adherence/Dropout	Frequency (Sessions/Week)	CG *	Design	Outcomes
Andersson et al. [13]	Sweden	63	71%	70.9	1	Local clubs (outdoor)	No info on dropout; regular participation	No info on dropout; regular participation	1	No	Cross-sectional	Mean HR ~78–80% HRmax; women had ↑ BMI and ↑ fat mass vs. men; both sexes within safe range.
Barbosa et al. [14]	Portugal	29	100%	64.5	12	Community (outdoor, adapted pitches)	Small-sided games (5–7 per team)	Median adherence 86.1% (77.8–97.2%); 2 dropouts	3	Yes	Quasi-experimental	↓ Abdominal fat, ↓ glucose, ↓ cholesterol; ↑ VO_2_peak ***
Capela et al. [15]	Portugal	50	100%	70.7	16	Not specified	Adapted matches (5–7 per team)	Mean adherence 81.6% ± 15.9%; dropout not relevant	3	Yes	RTC	↑ Muscle strength, ↑ balance, ↑ aerobic fitness; ↓ body fat.
Costa et al. [21]	Portugal	60	100%	67.0	2	Local pitches (outdoor)	5 × 5 (with and without goalkeeper)	No losses; all completed	1	No	RTC	With goalkeeper: ↑ HR **, ↑ distance covered; higher intensity vs. no goalkeeper
Duncan et al. [22]	United Kindom	43	100%	66.0	12	Community facility (indoor + synthetic outdoor pitch)	Small teams (~6 per team)	All completed; no dropouts	2	Yes	Case–control	Improvements: 30 s chair stand, TUG, 6 min walk test; No change in handgrip strength.
Egger et al. [23]	Germany	18	72%	69.0	12	Saarland University facilities (indoor)	Reduced matches (~5 per team)	Not applicable (acute study); no dropouts	2	No	Cross-sectional	↑ HR, ↑ RPE **** (moderate-to-vigorous intensity); mild DOMS reported; safe intervention with no dropouts
Harper et al. [24]	United Kindom	17	100%	66.0	8	Sports clubs (not specified)	Reduced matches, small teams	Not applicable; no dropouts	3	No	Cross-sectional	↑ Blood lactate, ↑ HR (76–84% HRmax), intensity classified as moderate-to-vigorous.

* CG: Control Group; ** HR: Heart Rate; *** VO_2_peak: Peak Oxygen Uptake; **** RPE: Rating of Perceived Exertion.

## Data Availability

Data sharing is not applicable.
